# Supplementary material for: Task rule and choice are reflected by layer-specific processing in rodent auditory cortical microcircuits
Source: Commun Biol. 2020 Jul 3;3:345. doi: 10.1038/s42003-020-1073-3 (PMC7335110; doi:10.1038/s42003-020-1073-3)
Supplement: Supplementary file 1 — Supplementary Information [file 42003_2020_1073_MOESM1_ESM.pdf]

Supplementary Information (Zempeltzi et al., 2020)

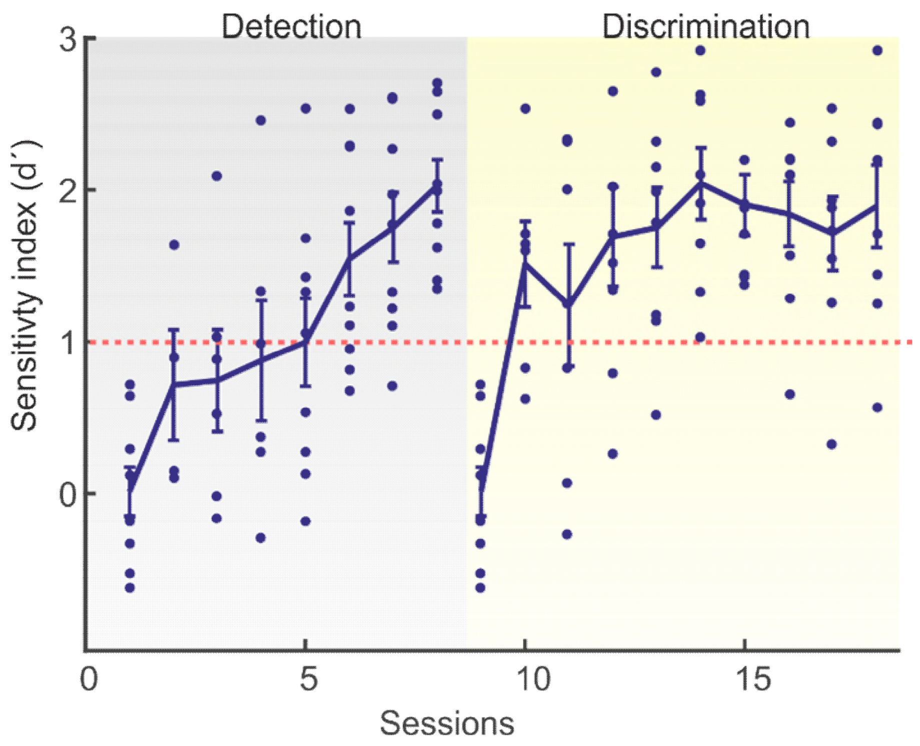

Supplementary Figure 1: ***Corresponding d'learning curves for the detection and discrimination phase.*** The d' learning curves were calculated based on conditioned response learning curves (Fig. 1b) for CS+ and CS– separately (hit rate = hits / number of CS+ trials; false alarm rates = false alarms / number of CS- trials). The sensitivity index d'allows to assess the behavioral sensitivity independent of experimental conditions biasing the response of the animal based on signal detection theory. For d' analysis during the detection phase, z-scores of corresponding hit rates were derived from the inverses of a standardized normal distribution function and divided by the inverses of spontaneous inter-trial shuttles ($d' = z_{hits} / z_{ITS}$). During discrimination, the d' was calculated as $d' = z_{hits} - z_{false\ alarms}$. Error bars indicate the standard error of mean (±s.e.m.). Single dots illustrate the single subject d' values per session.

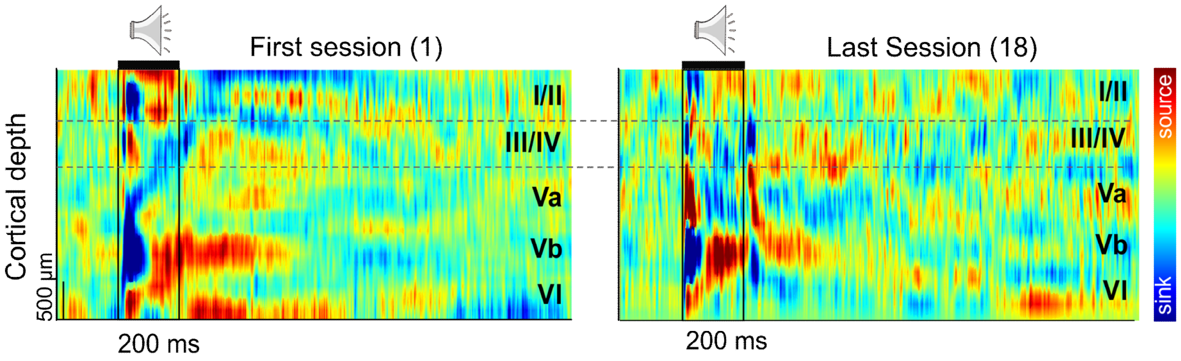

Supplementary Figure 2: ***Long-term stability CSD recordings from all cortical layers in A1.*** Representative example of an averaged CSD profile from one subject of the first training session (detection; *left*) and the last discrimination session, after two weeks (*right*). Based on the averaged auditory-evoked activity in response to the first presentation of the conditioned stimuli within a trial (time window: 1500ms; tone duration: 200 ms; indicated by the black frames) we assign the cortical input layers (I/II – VI) to the respective recording channels (indicated with the dashed lines). The example illustrates the stability of the electrode positioning over the course of the training.

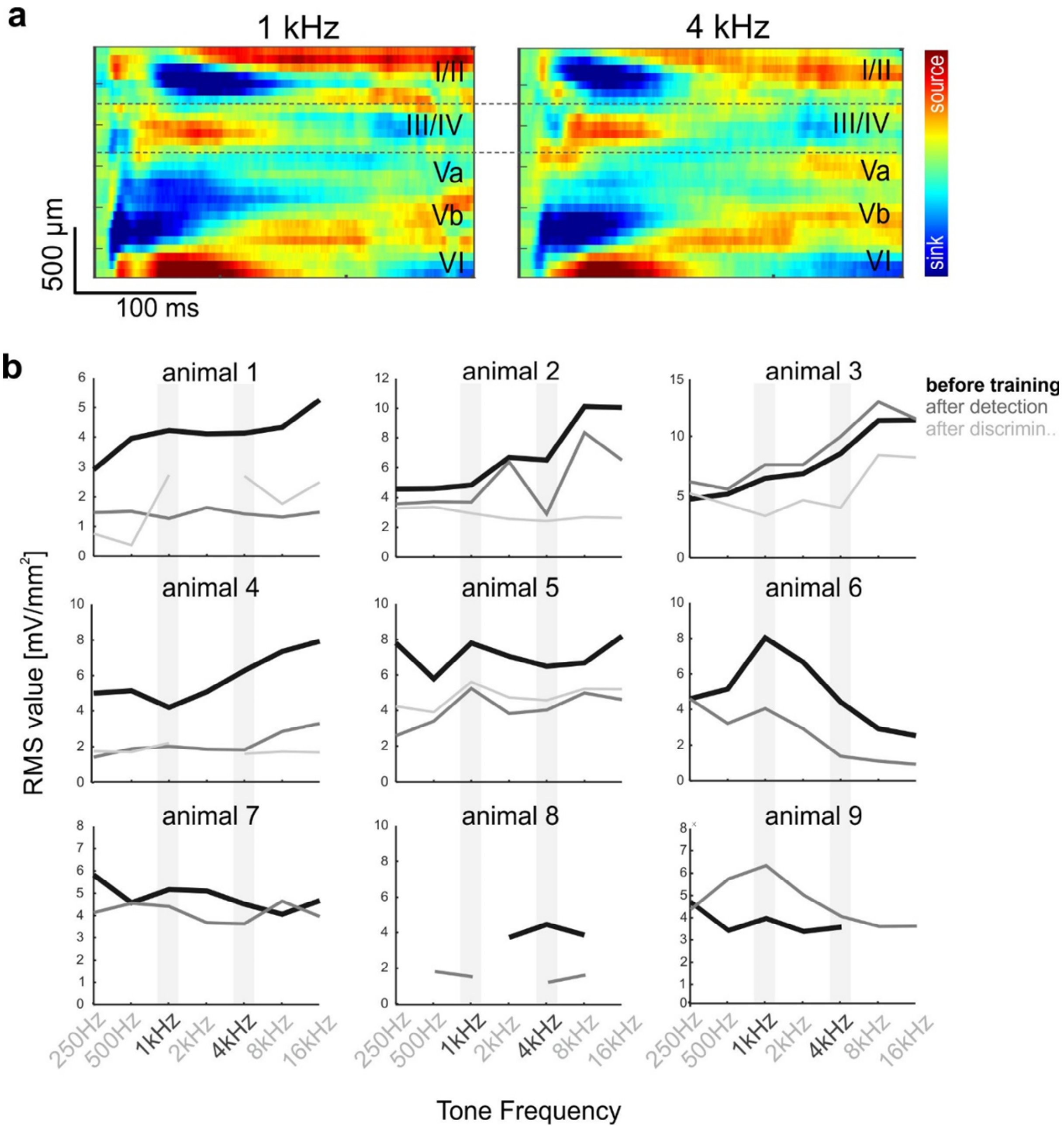

Supplementary Figure 3: ***Characterization of tuning properties in primary auditory cortex in recordings from individual animals.*** **a.** Representative example of an averaged CSD profile during the first awake, but passively listening measurement before the actual start of the behavioral training (see Methods and Materials). CSD activity is shown for the two pure tone frequencies also used during the later training, namely 1 kHz (*left*) and 4 kHz (*right;* tone duration: 200 ms, ISI 800 ms, 50 pseudorandomized repetitions, sound level 70 dB SPL). **b.** Individual tuning curves of mean CSD RMS values from dominant early synaptic inputs (averaged over 50 trials per frequency) are plotted as a function of stimulation frequency (n=9). We found a generally broad frequency tuning in awake, passively listening subject[1]. We repeated measuring passively recorded tuning curves after the consecutive detection and discrimination phase (grey curves). Associative training with two pure tone frequencies, hence, did not lead to systematic changes of the general tuning properties.

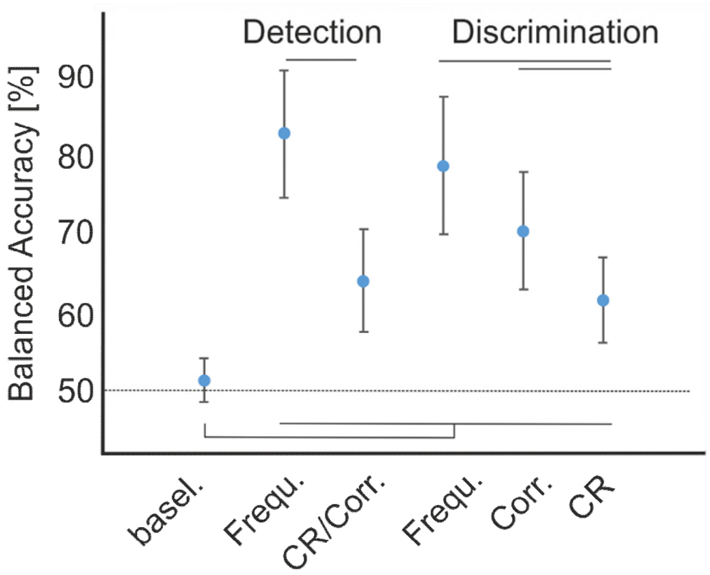

Supplementary Figure 4: ***Class separation based on linear support vector machine (SVM) classifiers.*** Linear support vector machine (SVM) classifiers (linear kernel; nested k-fold cross-validation of classifiers) were trained for targets reflecting either stimulus-related aspects of auditory processing or processing of task-dependent information. Individual SVM-based classification models were trained for bimodal targets reflecting either the stimulus-frequency (1kHz /4kHz), the behavioral choice (Go/NoGo), or the correctness of the choice option. Classifiers were trained on the entire CSD matrix in order to reveal class separation based on the columnar response. We chose time bins of 50ms before tone presentation (baseline) and directly after tone presentation to feed into the classifier. We built our class separation on balanced accuracy (BA) to consider that most targets were not balanced (i.e. correct vs incorrect trials). SVM-based class separation was significantly above chance level (50%) on all tested classes of stimulus frequency, the correctness of choice and the conditioned response during the detection or discrimination behavior and was higher compared to cortical baseline activity. Balanced accuracies (n=8; ±s.e.m.) are plotted for stimulus frequency, correctness of responses and conditioned responses. Note that correctness and conditioned response during the detection phase is the same (as a CR is always referred to as a hit). In all cases, we found the class separations to be significantly higher than 50% chance. Best accuracies were derived for the class ‘stimulus’, which corresponded to the 1 kHz or 4 kHz tone, yielding averaged accuracies across subjects of ~82% (detection) and ~78% (discrimination). Please note, during detection both tones were Go-stimuli, but had opposing contingencies during discrimination. Correspondingly, the classification of the target “correctness” during the detection phase separated trials with a conditioned response or not (hit vs. miss,; BA = ~64%). During discrimination, correctness was related to separate hits and correct rejection trials from false alarms and miss trials (BA = ~70%). A classifier trained on separating trials in which the animal showed a conditioned response or not during the discrimination phase was less accurate than on the target correctness during detection and discrimination and revealed significantly lower accuracies (BA = ~61%). Bars indicate Holm-corrected levels of significance tested by post-hoc Student’s t-tests.

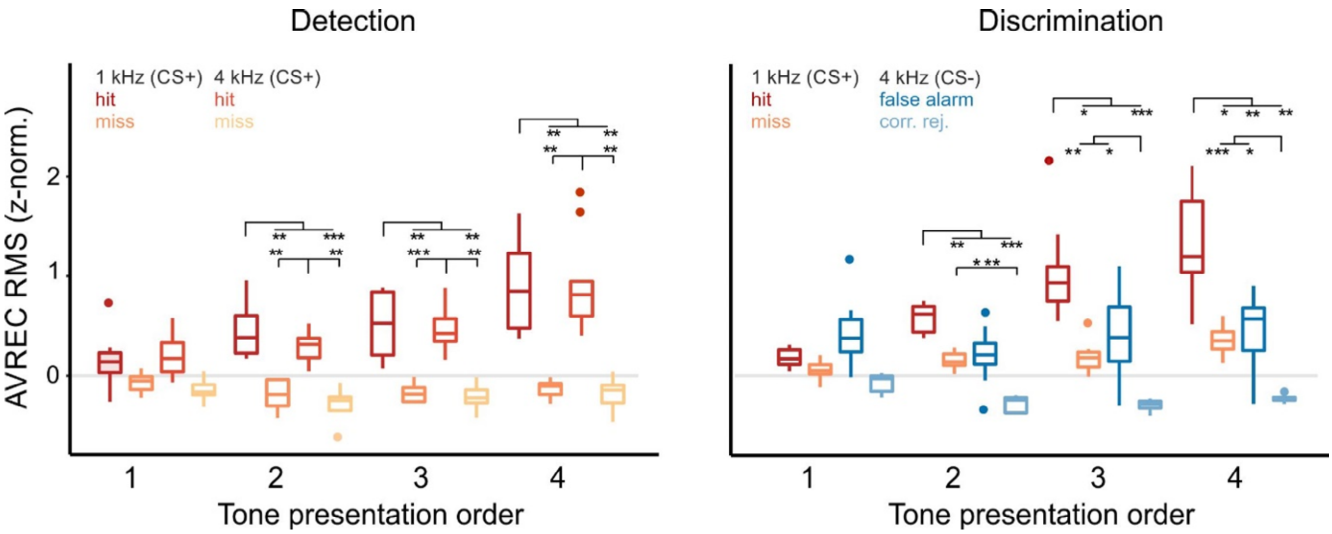

Supplementary Figure 5: ***Behavioral choices and contingency are represented in population activity of the A1 during time windows after the stimulus presentation.*** Averaged AVREC RMS values (at 500-1000 ms window after CS presentation) plotted with respect to the conditioned stimuli and behavioral choice. *Left*, During the detection phase evoked activity was significantly higher during hit trials compared to miss trials independent of the stimulation frequency (detection/ discrimination: n=9/8). *Right*, In the discrimination phase, cortical activity was strongest during correct hit trials and lowest during correct rejections. During trials of incorrect behavioral choices (miss/false alarm) tone-evoked activity was characterized by intermediate amplitudes and did not differ. These results show a very similar pattern with those from Figure 3. Box plots represent median (bar) and interquartile range, and bars represent full range of data. Dots represent outliers. Significant bars indicate differences revealed by a two-way rmANOVA and corresponding post-hoc tests with Holm-corrected levels of significance (see Supplementary Tab. 1b)

References

1. Deane, K. E. *et al.* Ketamine anesthesia induces gain enhancement via recurrent excitation in granular input layers of the auditory cortex. *J. Physiol.* (2020). doi:10.1113/jp279705

Suppl. Table 1a (cf. Fig 3) : rmANOVA of choice-related contingencies AVREC RMS

**Detection**			
Outcome	$F_{3,24}$ = 40.63	p < 0.001	η^2_{gen} = 0.66
Tone order	$F_{3,96}$ = 26.58	p < 0.001	η^2_{gen} = 0.25
Interaction	$F_{9,96}$ = 10.05	p < 0.001	η^2_{gen} = 0.27
Discrimination			
Outcome	$F_{3,21}$ = 31.67	p < 0.001	η^2_{gen} = 0.68
Tone order	$F_{3,84}$ = 26.39	p < 0.001	η^2_{gen} = 0.23
Interaction	$F_{9,84}$ = 17.52	p < 0.001	η^2_{gen}= 0.38

Suppl. Table 1b (cf. Supplementary Fig 5) : rmANOVA of choice-related contingencies AVREC RMS (time window 500-1000ms after CS presentation)

**Detection**			
Outcome	$F_{3,24}$ = 34.95	p < 0.001	η^2_{gen} = 0.60
Tone order	$F_{3,96}$ = 18.75	p < 0.001	η^2_{gen} = 0.23
Interaction	$F_{9,96}$ = 6.03	p < 0.001	η^2_{gen} = 0.22
Discrimination			
Outcome	$F_{3,21}$ = 30.82	p < 0.001	η^2_{gen} = 0.66
Tone order	$F_{3,84}$ = 19.88	p < 0.001	η^2_{gen} = 0.21
Interaction	$F_{9,84}$ = 10.28	p < 0.001	η^2_{gen}= 0.29

Suppl. Table 2 (cf. Fig 4B) : layer-wise GLMM applied to the conditioned stimuli 1 kHz vs 4 kHz

Detection '1 kHz vs 4 KHz'			
Layer I/II	R^2m = 0	R^2c = 0	p = 0.549
Layer III/IV	R^2m = 0	R^2c = 0	p = 0.649
Layer Va	R^2m = 0	R^2c = 0	p = 0.703
Layer Vb	R^2m = 0	R^2c = 0	p = 0.836
Layer VI	R^2m = 0	R²c = 0	p = 0.754
Discrimination '1 kHz vs 4 KHz'			
Layer I/II	R^2m = 0.065	R^2c = 0.080	p < 0.001
Layer III/IV	R^2m = 0.121	R^2c = 0.219	p < 0.001
Layer Va	R^2m = 0.095	R^2c = 0.175	p < 0.001
Layer Vb	R^2m = 0.076	R^2c = 0.118	p < 0.001
Layer VI	R^2m = 0.034	R^2c = 0.058	p = 0.001

uppl. Table 3 (cf. Fig 5B) : layer-wise GLMM applied to the behavioral choices

Detection 'Hit vs Miss'			
Layer I/II	R^2m = 0.113	R^2c = 0.537	p = 0.056
Layer III/IV	R^2m = 0.190	R^2c = 0.406	p < 0.001
Layer Va	R^2m = 0.242	R^2c = 0.512	p < 0.001
Layer Vb	R^2m = 0.258	R^2c = 0.569	p < 0.001
Layer VI	R^2m = 0.200	R^2c = 0.396	p < 0.001
Discrimination 'Hit vs Miss'			
Layer I/II	R^2m = 0.144	R^2c = 0.240	p < 0.001
Layer III/IV	R^2m = 0.188	R^2c = 0.361	p = 0.001
Layer Va	R^2m = 0.086	R^2c = 0.214	p = 0.216
Layer Vb	R^2m = 0.049	R^2c = 0.152	p = 0.001
Layer VI	R^2m = 0.033	R^2c = 0.212	p = 0.118
Discrimination 'False alarm vs Correct Rejection'			
Layer I/II	R^2m = 0.164	R^2c = 0.465	p < 0.001
Layer III/IV	R^2m = 0.170	R^2c = 0.318	p < 0.001
Layer Va	R^2m = 0.118	R^2c = 0.234	p < 0.001
Layer Vb	R^2m = 0.105	R^2c = 0.263	p < 0.001
Layer VI	R^2m = 0.096	R^2c = 0.239	p < 0.001

Suppl. Table 4 (cf. Fig 6B) : layer-wise GLMM applied to the choice accuracy

Discrimination 'Hit vs Correct rejection'			
Layer I/II	R^2m = 0.517	R^2c = 0.607	p < 0.001
Layer III/IV	R^2m = 0.435	R^2c = 0.573	p < 0.001
Layer Va	R^2m = 0.38	R^2c = 0.509	p < 0.001
Layer Vb	R^2m = 0.378	R^2c = 0.479	p < 0.001
Layer VI	R^2m = 0.193	R^2c = 0.287	p < 0.001
Discrimination 'Miss vs False Alarm'			
Layer I/II	R^2m = 0.028	R^2c = 0.184	p = 0.004
Layer III/IV	R^2m = 0.049	R^2c = 0.202	p = 0.047
Layer Va	R^2m = 0.039	R^2c = 0.225	p = 0.058
Layer Vb	R^2m = 0	R^2c = 0.21	p = 0.98
Layer VI	R^2m = 0.015	R^2c = 0.178	p = 0.322
